# A decade of inequality in maternity care: antenatal care, professional attendance at delivery, and caesarean section in Bangladesh (1991–2004)

**DOI:** 10.1186/1475-9276-6-9

**Published:** 2007-08-30

**Authors:** Simon M Collin, Iqbal Anwar, Carine Ronsmans

**Affiliations:** 1 Department of Social Medicine, University of Bristol, Canynge Hall, Whiteladies Road, Bristol, BS8 2PR, UK; 2 Public Health Sciences Division, Reproductive Health Unit, ICDDR-B, 68 Shahid Tajuddin Ahmed Sharani, Mohakhali (GPO Box 128, Dhaka 1000), Dhaka 1212, Bangladesh; 3 Maternal Health Group, Department of Epidemiology & Population Health, London School of Hygiene & Tropical Medicine, Keppel Street, London, WC1E 7HT, UK

## Abstract

**Background:**

Bangladesh is committed to the fifth Millennium Development Goal (MDG-5) target of reducing its maternal mortality ratio by three-quarters between 1990 and 2015. Since the early 1990s, Bangladesh has followed a strategy of improving access to facilities equipped and staffed to provide emergency obstetric care (EmOC).

**Methods:**

We used data from four Demographic and Health Surveys conducted between 1993 and 2004 to examine trends in the proportions of live births preceded by antenatal consultation, attended by a health professional, and delivered by caesarean section, according to key socio-demographic characteristics.

**Results:**

Utilization of antenatal care increased substantially, from 24% in 1991 to 60% in 2004. Despite a relatively greater increase in rural than urban areas, utilization remained much lower among the poorest rural women without formal education (18%) compared with the richest urban women with secondary or higher education (99%). Professional attendance at delivery increased by 50% (from 9% to 14%, more rapidly in rural than urban areas), and caesarean sections trebled (from 2% to 6%), but these indicators remained low even by developing country standards. Within these trends there were huge inequalities; 86% of live births among the richest urban women with secondary or higher education were attended by a health professional, and 35% were delivered by caesarean section, compared with 2% and 0.1% respectively of live births among the poorest rural women without formal education. The trend in professional attendance was entirely confounded by socioeconomic and demographic changes, but education of the woman and her husband remained important determinants of utilization of obstetric services.

**Conclusion:**

Despite commendable progress in improving uptake of antenatal care, and in equipping health facilities to provide emergency obstetric care, the very low utilization of these facilities, especially by poor women, is a major impediment to meeting MDG-5 in Bangladesh.

## Background

The target of the fifth Millennium Development Goal (MDG-5) is a 75% reduction in maternal mortality between 1990 and 2015 [1]. Current trends indicate that this target is unlikely to be met in many countries, particularly in sub-Saharan Africa and south Asia [2,3]. Skilled attendance at birth, backed-up by access to referral-level facilities, is the key strategy to achieve MDG-5. In most low-income countries, huge disparities exist between women from poor and rich households in their ability to access professional delivery care [4,5]. There is an urgent need to develop effective and affordable programmes that make skilled delivery care, including emergency obstetric care, accessible to all women.

Bangladesh has seen a gradual decline in its maternal mortality ratio (deaths per 100,000 live births) over the past decade, from 500 in 1990 to 400 in 2001, [6] but the ratio remains unacceptably high, representing 12,000 maternal deaths per year. The official MDG-5 target of 143 deaths/100,000 live births by 2015 can only be achieved by overcoming gender and socioeconomic inequalities, and cultural barriers, which prohibit access to skilled and emergency obstetric care for the vast majority of Bangladeshi women [6-8].

Programmes implemented by the Government of Bangladesh to reduce maternal mortality have followed the evolving strategies of the Safe Motherhood movement [9]. Since the early 1990s, the emphasis has been on improving the provision of emergency obstetric care (EmOC). This strategy has been reasonably successful in upgrading health facilities and training staff to provide basic and comprehensive emergency care, [6,10-12] but the key question is whether there has been a corresponding uptake of these services by women across all strata of Bangladeshi society.

This paper attempts to answer this question by examining trends in three key maternal health indicators; antenatal care, delivery attended by a health professional (doctor, nurse, or midwife), and delivery by caesarean section. The latter indicates whether the EmOC strategy has translated into better access to emergency obstetric care; while utilization of antenatal and professional delivery care illustrate the existence of a broader support strategy for women during pregnancy and birth. Trends were examined to determine whether they were distributed proportionately between socio-economic groups, which would indicate that the strategy has reached all sections of society.

## Methods

This study is based on the 1993–1994, 1996–1997, 1999–2000, and 2004 Bangladesh Demographic and Health Surveys (DHS) [8,13-15]. The four DHS datasets were combined to obtain birth-based trends in obstetric care over the period 1991–2004; in the 1993–1994 survey women were asked questions relating to live births in the preceding three years; in the 1996–1997, 1999–2000 and 2004 surveys women were asked questions relating to live births in the preceding five years.

The analyses focused on whether women had received one or more antenatal consultations from a health professional (doctor, nurse, or midwife – excluding trained or untrained traditional birth attendants), whether a health professional was present at delivery, and whether the birth occurred at home or in a health facility. The 1999–2000 and 2004 surveys also reported whether the delivery was by caesarean section. The other variables used in the analyses were twin birth, mother's age at delivery (in five year age groups), parity (1, 2, 3, 4, 5+), mother's highest level of education (none, primary, secondary, higher), father's highest level of education, and area of residence (rural/urban and administrative division).

To measure socioeconomic status, asset quintiles were computed using the principal components analysis method of Filmer and Pritchett [16]. The principal components analysis was conducted using the pooled household-level DHS data, which were representative of the entire Bangladeshi population between 1991 and 2004. Asset variables available in each DHS dataset were used: source of drinking water; type of latrine; principal material of floor, wall, and roof; electricity supply; ownership of radio, television or bicycle. The asset quintiles were derived from the first principal component.

Changes over time in the socio-demographic profile of the population were examined by analysing changes in the socio-demographic characteristics of the population over 3-year time periods (except for 2000–2004 which covered 4 years). Logistic regressions were used to calculate crude and adjusted odds ratios for each outcome, using Wald tests to assess statistical significance, taking into account survey design (sampling weights and strata) and clustering (to account for women who contributed more than one live birth in the preceding three/five years). Crude associations between antenatal care and delivery care with a health professional or by caesarean section were analysed, but the multivariate models for trends in professional attendance and caesarean section did not include antenatal care as a predictor variable because antenatal care was considered to be on the causal pathway.

Time trend interactions with asset quintile and urban/rural residence were explored to determine whether the odds of antenatal care and delivery with a skilled attendant or by caesarean section were changing at the same rate in all socio-economic groups and in urban and rural areas. Because very few caesarean sections occurred among women in the bottom economic quintiles, this interaction was modelled in two strata only, comparing trends in the middle and top two quintiles with trends in the bottom two quintiles. All analyses were performed using Stata v8 (StataCorp. 2003. *Stata Statistical Software: Release 8*. College Station, TX: StataCorp LP).

## Results

### Trends in the socio-demographic profile of the population

There were major demographic and socio-economic changes between 1991 and 2004 (Table 1). The proportion of women living in urban areas doubled from 13.4% in 1991–93 to 30.0% in 2000–04, while the proportion of women with no formal education decreased from 56.5% to 35.8%. In 1991–93, 27.6% of women lived in households in the lowest asset quintile, compared with 10.2% in 2000–04. Fertility declined dramatically; women reporting five or more children declined from 35.9% in 1991–93 to 17.9% in 2000–04. The distribution of assets indicated extreme inequality: none of the households in the bottom quintile had electricity or owned a radio, and only 4% owned a bicycle; even in the second highest quintile, only half had electricity or a radio; in the top quintile, 86% had electricity and 63% owned a television (data not shown).

**Table 1:** Trends in socio-demographic characteristics in Bangladesh (1991–2004)

**Socioeconomic and demographic variables**	**Year**				
		
	**1991–1993 (N = 6,221)**	**1994–1996 (N = 6,312)**	**1997–1999 (N = 5,255)**	**2000–2004 (N = 5,965)**	P-value*
**Area of residence**					P < 0.001
Rural	86.6%	82.1%	73.8%	70.0%	
Urban	13.4%	17.9%	26.2%	30.0%	

**Maternal education**					P < 0.001
No education	56.5%	52.4%	44.3%	35.8%	
Primary	28.2%	28.4%	29.0%	31.1%	
Secondary	13.4%	16.2%	22.2%	27.0%	
Higher	1.8%	3.0%	4.5%	6.0%	

**Paternal education**					P < 0.001
No education	47.4%	46.0%	42.0%	38.2%	
Primary	24.8%	25.7%	25.3%	26.8%	
Secondary	20.5%	19.6%	22.4%	24.5%	
Higher	7.4%	8.6%	10.3%	10.5%	

**Asset quintiles**					P < 0.001
Poorest	27.6%	21.6%	17.4%	10.2%	
Poor	24.5%	22.4%	19.2%	15.9%	
Middle	19.5%	19.2%	18.6%	22.5%	
Richer	15.1%	19.0%	21.7%	24.9%	
Richest	13.3%	17.8%	23.2%	26.5%	

**Age (years)**					P < 0.001
10–19	13.8%	15.7%	18.3%	18.3%	
20–24	33.0%	30.8%	31.1%	34.2%	
25–29	28.3%	27.8%	26.9%	23.9%	
30–34	13.8%	15.6%	14.7%	14.5%	
35–39	7.6%	6.8%	6.3%	6.5%	
40–49	3.5%	3.3%	2.8%	2.6%	

**Parity**					P < 0.001
1	9.1%	12.2%	16.8%	23.0%	
2	18.6%	22.6%	25.8%	26.8%	
3	20.0%	20.5%	20.3%	19.5%	
4	16.4%	15.1%	13.7%	12.9%	
≥5	35.9%	29.6%	23.4%	17.9%	

**Total**	100%	100%	100%	100%	

* P-values from Chi-squared test for differences in proportions by year group

### Trends in antenatal consultations and professional attendance at birth

**Figure 1:**
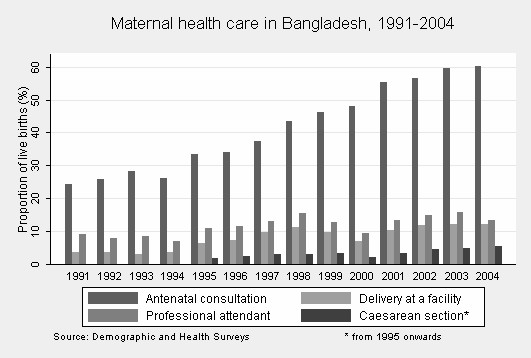
Maternal health care in Bangladesh, 1991–2004.

The proportion of women who had at least one antenatal consultation increased dramatically from 24.4% in 1991 to 60.3% in 2004 (crude OR 1.14 per year, 95% CI 1.13–1.15) (Figure 1, Table 2). In contrast, the proportion of deliveries attended by a professional increased only marginally from 9.2% in 1991 to 13.5% in 2004 (crude OR 1.06 per year, 95% CI 1.05–1.07) (Figure 1, Table 2). The entire increase in professional attendance occurred in health facilities, even though most women still give birth at home (data not shown).

**Table 2:** Determinants of antenatal care and professional attendance at delivery in Bangladesh (1991–2004)

	**Antenatal care consultation (one or more)**					**Professional attendance at delivery**				
	
**Live births (N = 23,769)**	**%**	**Univariate analysis**		Multivariate analysis*		**%**	**Univariate analysis**		Multivariate analysis*	
		**OR**	**(95% CI)**	**OR**	**(95% CI)**		**OR**	**(95% CI)**	**OR**	**(95% CI)**
**Year groups**										
1991 – 1993	26.6	1.00		1.00		8.4	1.00		1.00	
1994 – 1996	31.7	1.28	(1.18, 1.39)	1.09	(1.00, 1.20)	10.3	1.24	(1.10, 1.42)	0.90	(0.78, 1.05)
1997 – 1999	43.0	2.08	(1.91, 2.27)	1.48	(1.34, 1.64)	13.6	1.71	(1.50, 1.95)	0.95	(0.82, 1.11)
2000 – 2004	55.8	3.48	(3.19, 3.79)	2.52	(2.28, 2.78)	13.4	1.68	(1.48, 1.91)	0.96	(0.82, 1.12)

**Annual trend**										
1991 – 2004	38.3	1.14	(1.13, 1.15)	1.11	(1.10, 1.12)	11.3	1.06	(1.05, 1.07)	1.00	(0.99, 1.02)

**Asset quintile**										
Lowest	20.6	1.00		1.00		3.1	1.00		1.00	
Second	24.8	1.27	(1.13, 1.42)	1.04	(0.92, 1.17)	3.8	1.24	(0.96, 1.58)	0.99	(0.76, 1.28)
Middle	33.7	1.96	(1.76, 2.20)	1.27	(1.12, 1.44)	5.8	1.90	(1.50, 2.40)	1.17	(0.91, 1.51)
Fourth	46.5	3.35	(3.01, 3.74)	1.60	(1.41, 1.82)	11.5	4.03	(3.26, 5.00)	1.72	(1.36, 2.18)
Highest	70.8	9.37	(8.36, 10.50)	2.59	(2.24, 2.99)	35.3	16.92	(13.85, 20.67)	3.06	(2.40, 3.90)

**Woman's education**										
None	23.6	1.00		1.00		4.3	1.00		1.00	
Primary	39.0	2.09	(1.93, 2.26)	1.48	(1.35, 1.62)	8.3	2.02	(1.75, 2.33)	1.29	(1.09, 1.52)
Secondary	65.7	6.10	(5.58, 6.67)	2.51	(2.22, 2.83)	23.8	7.00	(6.15, 7.98)	2.15	(1.79, 2.59)
Higher	94.8	52.89	(37.27, 75.05)	9.97	(6.86, 14.47)	66.8	44.99	(36.95, 54.77)	5.31	(4.05, 6.95)

**Husband's education**										
None	25.8	1.00		1.00		4.5	1.00		1.00	
Primary	35.7	1.59	(1.47, 1.73)	1.14	(1.04, 1.26)	7.1	1.61	(1.38, 1.88)	1.07	(0.90, 1.28)
Secondary	52.9	3.13	(2.88, 3.41)	1.40	(1.25, 1.55)	17.3	4.43	(3.87, 5.08)	1.61	(1.36, 1.92)
Higher	78.1	9.59	(8.40, 10.95)	1.79	(1.50, 2.13)	45.3	17.62	(15.22, 20.40)	2.41	(1.94, 3.00)

**Area of residence**										
Rural	32.9	1.00		1.00		7.2	1.00		1.00	
Urban	66.1	3.98	(3.67, 4.31)	1.97	(1.79, 2.18)	31.8	6.06	(5.50, 6.68)	2.70	(2.39, 3.06)

* Multivariate model controls for all variables in table plus age, parity, singleton birth, and administrative division.

**Figure 2:**
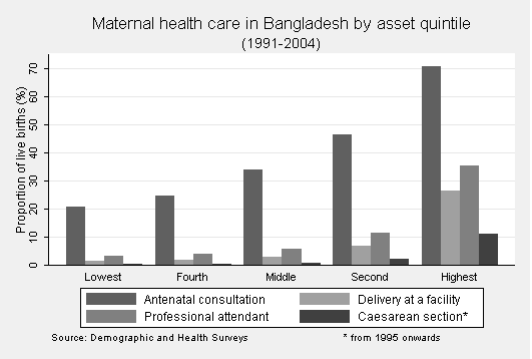
Maternal health care in Bangladesh by asset quintile (1991–2004).

Antenatal care and professional attendance at birth were more common among urban, rich and educated women (Table 2, Figure 2). About two-thirds of urban women had an antenatal visit, compared to only one third of rural women (crude OR 3.98, 95% CI 3.67–4.31). Urban/rural and poor/rich disparities in professional attendance at birth were also substantial, although the coverage remained low even for the urban and more affluent sections of society: the proportion of births attended by a professional was only 35% among the richest and 32% among urban women.

Disparities become more pronounced at the extremes of the socio-economic spectrum (Table 3). Almost all (98.8%) urban women with secondary or higher education in the highest asset quintile made one or more antenatal visits, compared with less than one fifth (17.5%) of rural, uneducated women in the lowest asset quintile. Similarly, 86.1% of secondary or more highly educated, urban women in the highest quintile gave birth with a professional; among uneducated, rural women in the lowest quintile, this proportion was 2.2%.

**Table 3:** Inequality in maternity care: summary measures for the period 1991–2004 at opposite ends of the socioeconomic spectrum

**Proportion of births at which each aspect of maternity care was accessed**	**Antenatal consultation**	**Professional attendance**	Caesarean section*
**Urban women with secondary or higher education in the highest asset quintile**	98.8%	86.1%	34.8%
**Rural women with no education in the lowest asset quintile**	17.5%	2.2%	0.1%

* 1995–2004

The trends over time in antenatal care persisted after adjusting for socio-demographic variables (adjusted OR per year 1.11 95% CI 1.10–1.12), but they disappeared for professional attendance at birth (adjusted OR per year 1.00 95% CI 0.99–1.02) (Table 2). This suggests that socioeconomic changes (an increasingly urban, wealthier and more educated population) fully explain the small increase in professional attendance over time.

Antenatal care was strongly associated with professional attendance. The percent of births with a professional attendant was 3.6% for mothers with no antenatal consultations, compared with 24.1% for mothers with at least one consultation (crude OR = 8.58 (95% CI 7.65–9.64)). The odds of professional attendance increased in quadratic fashion according to the number of antenatal consultations; 9.4%, 15.7%, 22.1%, and 34.8% of mothers with one, two, three, or four consultations had a professional birth attendant. For women who had ten or more consultations, 74.9% had a professional birth attendant. Among mothers who gave birth with a professional attendant, 80.8% had one or more antenatal consultations, compared with 32.8% of mothers who had no professional attendance at delivery.

### Trends in caesarean sections

Caesarean section rates rose steadily over time (from 1.9% in 1995 to 5.6% in 2004, crude OR per year 1.11 95% CI 1.06–1.16) (Figure 1, Table 4). The poor-rich gap in caesarean sections was huge: the rate was less than 1% in each of the three bottom quintiles (representing 60% of women), compared with 11.1% in the top quintile (representing 20% of women) (Figure 2). The caesarean section rate for urban women educated to secondary or higher in the richest quintile was 34.8%; the rate was 0.1% for mothers who were uneducated, from rural areas, and in the bottom quintile (Table 3). Trends over time in caesarean sections became somewhat more pronounced after adjusting for covariates, and socio-economic characteristics remained independent predictors. Antenatal care was strongly associated with caesarean section: among mothers who made no antenatal visit the caesarean section rate was only 0.3% while the rate was 1.4%, 3.8%, 4.3%, and 18.8% for mothers who made one, two, three, or four or more antenatal visits respectively.

**Table 4:** Determinants of delivery by caesarean section in Bangladesh (1995–2004)

	**Caesarean section**				
	
**Live births (N = 13,714)**	**%**	**Univariate analysis**		Multivariate analysis*	
		**OR**	**(95% CI)**	**OR**	**(95% CI)**
**Year**					
1995 – 1996	2.1	1.00			
1997 – 1999	3.2	1.53	(1.10, 2.12)	1.71	(1.18, 2.48)
2000 – 2004	3.8	1.83	(1.32, 2.55)	2.24	(1.55, 3.25)

**Annual trend**					
1991 – 2004	3.3	1.11	(1.06, 1.16)	1.14	(1.09, 1.19)

**Asset quintile**					
Lowest	0.3	1.00			
Second	0.4	1.54	(0.52, 4.51)	1.17	(0.39, 3.48)
Middle	0.7	2.73	(1.02, 7.26)	1.56	(0.58, 4.22)
Fourth	2.2	8.48	(3.55, 20.31)	3.24	(1.30, 8.11)
Highest	11.1	46.30	(20.09, 106.72)	6.74	(2.69, 16.84)

**Woman's education**					
None	0.6	1.00			
Primary	1.5	2.55	(1.57, 4.14)	1.45	(0.85, 2.46)
Secondary	5.9	10.36	(6.95, 15.45)	2.36	(1.37, 4.06)
Higher	26.3	59.16	(39.05, 89.64)	4.00	(2.16, 7.40)

**Husband's education**					
None	0.8	1.00			
Primary	1.2	1.62	(1.01, 2.59)	0.84	(0.51, 1.40)
Secondary	4.8	6.68	(4.47, 9.98)	1.54	(0.93, 2.57)
Higher	16.4	26.06	(17.74, 38.28)	1.90	(1.08, 3.34)

**Area of residence**					
Rural	1.6	1.00			
Urban	9.0	6.02	(4.80, 7.57)	2.02	(1.53, 2.67)

*Multivariate model controls for all variables in table plus age, parity, singleton birth, and administrative division.

### Time trends in antenatal consultations, attendant at birth and caesarean sections by asset quintile and urban/rural residence

Univariate analyses of interaction suggested more rapid rises in antenatal care and professional attendance in rural compared with urban areas (p = 0.009 and p < 0.001 respectively), but time trends did not vary by asset quintile (Table 5). Neither of these interactions was evident in multivariate models. There was no evidence for variation in caesarean section trends by rural or urban residence, or by asset quintile, though the rise in caesarean sections was only seen among women in the middle and two upper quintiles (Table 5).

**Table 5:** Annual trends in maternity care indicators in Bangladesh (1991–2004) stratified by asset quintile and urban/rural area of residence

	**Antenatal consultation (1991–2004)**		**Professional attendance (1991 – 2004)**		**Caesarean section (1995 – 2004)**	
	
	**OR**	**(95% CI)**	**OR**	**(95% CI)**	**OR**	**(95% CI)**
**Asset quintile (univariate analysis)**						
Lowest quintile	1.13	(1.11, 1.16)	1.01	(0.95, 1.07)	1.02	(0.88, 1.18)
Second quintile	1.13	(1.11, 1.16)	1.04	(0.99, 1.08)		
Middle quintile	1.11	(1.09, 1.13)	1.02	(0.98, 1.06)	1.31	(1.03, 1.67)
Fourth quintile	1.13	(1.11, 1.15)	1.04	(1.01, 1.07)	1.30	(1.16, 1.46)
Highest quintile	1.09	(1.07, 1.12)	1.01	(0.99, 1.03)	1.07	(1.02, 1.13)
P-value*	0.180		0.627		0.114	

**Residence (univariate analysis)**						
Rural	1.14	(1.13, 1.15)	1.06	(1.04, 1.08)	1.11	(1.03, 1.20)
Urban	1.09	(1.07, 1.11)	0.99	(0.97, 1.01)	1.15	(1.09, 1.21)
P-value**	0.009		<0.001		0.505	

* Wald-test for interaction between year group and asset quintile

** Wald-test for interaction between year group and area of residence

## Discussion

This study has revealed important upward trends for antenatal care in Bangladesh between 1991 and 2004, but progress for deliveries attended by health care professionals was disappointingly small. This latter finding brings up-to-date an earlier analysis of trends in delivery care in Bangladesh which was based on the first three DHS [17]. Births with a professional increased from an extremely low level of 9% in the early nineties to a mere 13% in the new Millennium, though near universal coverage was reached among urban, wealthy and highly educated women. Births by caesarean also rose, but this effect was only seen among wealthier women. A comparison of the extreme ends of the social spectrum revealed huge gaps in access to caesarean sections, with the urban rich and educated having excessive caesareans (at a rate of 34.8%) compared to the rural uneducated poor among whom caesarean sections were almost non-existent (at a rate of 0.1%).

The limited progress towards increasing skilled attendance at birth is perhaps not surprising, given the focus of the national programme on upgrading EmOC facilities rather than training and deploying midwives. Wider provision of EmOC has clearly increased access to obstetric surgery, although much of the increase occurred in the private sector (data not shown), and only the wealthier women benefit from these interventions. The majority of caesarean sections in Bangladesh are performed at private facilities, [18] and whether the increasing caesarean section rates reflect actual gains towards meeting the need for obstetric care is difficult to say. All-cause caesareans may comprise women who need a surgical intervention in order to save their or their baby's life as well as women for whom there is no clinical need, hence interpreting crude caesarean rates is difficult [19]. Even though not all caesarean sections are necessarily life-saving, caesarean section rates of less than 1% indicate an unmet need for potentially life-saving care [20,21]. Data from the rural Matlab area of Bangladesh suggest that the met need for obstetric care is increasing, [22] but whether this is true at national level is not known.

The Government of Bangladesh has set a specific MDG target to increase skilled attendance at birth to 50% by 2010. With the current rate of progress, this target will not be reached, and the challenges are huge. The density of midwives was estimated at 1.8 midwives per 10,000 population in 2004 [23]. This falls within the range reported for neighbouring Malaysia and Sri Lanka (3.4/10000 and 1.6/10000 respectively), countries which have rates of skilled attendance above 97% [24]. However, in urban areas, where the density of midwives is likely to be higher and where other physical barriers such as transport are unimportant, skilled attendance is still only 32%, suggesting that supply alone is not the solution. Women in Bangladesh may have a strong attachment to home-based birth traditions, and may prefer traditional birth attendants with whom they have closer social links [25]. Similarly for EmOC; our findings suggest that the problem is not one of upgrading facilities, but rather in persuading women to use these facilities.

Financial barriers to utilization of facility-based care are prohibitive among the poor, even where the actual care is free-of-charge [26-29]. The introduction of a maternal health voucher scheme for poor mothers in 21 sub-districts of Bangladesh is certainly a great step forward, but the challenges in implementing and scaling-up this type of demand-side financing program are considerable [30]. Removing financial barriers alone will not eliminate the poor-rich gap, and further strengthening of the demand side will be necessary [31,32].

The levels of maternal mortality in Bangladesh are remarkably low given the extremely low levels of uptake of maternity care. The maternal mortality ratio has been estimated at between 320 and 400 per 100,000 live births, and it may have fallen since the late 1980s [6]. The reasons for this fall remain a puzzle. A decreasing trend of maternal mortality with corresponding increases in population based caesarean section rates, and without increase of professional attendance at birth, support the views expressed by Maine and others that the accessibility of EmOC plays a large role in shaping maternal outcomes exclusive of the use of skilled attendance at births [33]. This together with a reduction in abortion mortality, lower fertility and general improvements in health may explain part of the decline [22].

The study has some limitations. First, data on area of residence in the three DHS surveys were not strictly comparable because, unlike the 1999–2000 survey, the 1993–1994 and 1996–1997 surveys categorized "other urban" areas as "rural". Second, the recall periods were different; 3 years for the first survey, 5 years for the other surveys. This issue, along with that of birth-based versus woman-based analyses, is explored in detail by Bell et al [17]. We decided that the disadvantage of the difference in recall error between the oldest survey and the other three surveys would be more than offset by the gain in power obtained by aggregating all available data. We concurred with Bell et al in choosing a birth-based analysis, despite the bias which this denominator introduces due to over-representation of poorer women who tend to have more children [17]. Third, the construction of a pooled asset score over a 16-year period, ignoring the possibly changing value of assets over time, may have led to misclassification of socio-economic status. Some authors have suggested that the ranking of households is robust to the asset items included, [16] while others have suggested the opposite [34]. However, the asset score showed surprisingly good discriminatory power to reveal inequalities in birth attendance and caesarean sections, and the changes in the distribution of wealth groups over time is consistent with the rapid economic growth in Bangladesh during this time. The inclusion of other important determinants of access to health care, in particular maternal education, in the model in conjunction with the asset scores, would be expected to give a reasonably accurate picture of inequalities within Bangladesh [35].

## Conclusion

The central finding of this study is the poor coverage for professional delivery care services and existence of pronounced socioeconomic inequities in utilization of maternal health care services. In striving to achieve national average targets such as Millennium Development Goals, the reduction of socioeconomic inequalities in maternal health should be viewed as a central policy and programme goal. Barriers to health care access are well known, and a greater focus is now needed on implementing and evaluating interventions that benefit the poor, particularly in rural areas.

## Competing interests

The authors declare that they have no competing interests. Simon Collin and Carine Ronsmans were funded by the London School of Hygiene and Tropical Medicine, the UK Department for International Development, and IMMPACT (Initiative for Maternal Mortality Programme Assessment, funded by the Bill & Melinda Gates Foundation, the Department for International Development, the European Commission and USAID). Iqbal Anwar was funded by the ICDDR, B and USAID. The funders have no responsibility for the information provided or views expressed in this manuscript. The views expressed herein are solely those of the authors.

## Authors' contributions

CR raised the research question and coordinated research activities. SC and IA analysed the data. All authors contributed to early drafts of the manuscript, and all authors read and approved the final manuscript.

## References

[B1] (2006). The Millennium Development Goals Report.

[B2] Campbell OM, Graham WJ (2006). Strategies for reducing maternal mortality: getting on with what works. Lancet.

[B3] Koblinsky M, Matthews Z, Hussein J, Mavalankar D, Mridha MK, Anwar I, Achadi E, Adjei S, Padmanabhan P, van Lerberghe W (2006). Going to scale with professional skilled care. Lancet.

[B4] Gwatkin DR, Bhuiya A, Victora CG (2004). Making health systems more equitable. Lancet.

[B5] Kunst AE, Houweling T (2001). A global picture of poor-rich differences in the utilisation of delivery care.. Studies in Health Services Organisation and Policy.

[B6] (2003). Bangladesh Maternal Health Services and Maternal Mortality Survey 2001.

[B7] (2005). Millennium Development Goals: Bangladesh Progress Report.

[B8] (2005). Bangladesh Demographic and Health Survey 2004.

[B9] Huque ZA, Leppard M, Mavalanker D, Akhter HH, Chowdhury TA, Berer M, Sundari Ravindran TK (1999). Safe Motherhood Programmes in Bangladesh. Safe motherhood initiatives: critical issues.

[B10] Gill Z, Ahmed JU (2004). Experience from Bangladesh: implementing emergency obstetric care as part of the reproductive health agenda. Int J Gynaecol Obstet.

[B11] Islam MT, Hossain MM, Islam MA, Haque YA (2005). Improvement of coverage and utilization of EmOC services in southwestern Bangladesh. Int J Gynaecol Obstet.

[B12] Islam MT, Haque YA, Waxman R, Bhuiyan AB (2006). Implementation of emergency obstetric care training in Bangladesh: lessons learned. Reprod Health Matters.

[B13] (2001). Bangladesh Demographic and Health Survey, 1999-2000.

[B14] Mitra SN, Al-Sabir A, Cross AR, Jamil K (1997). Bangladesh Demographic and Health Survey, 1996-1997.

[B15] Mitra SN, Nawab AM, Islam S, Cross AR, Saha T (1994). Bangladesh Demographic and Health Survey, 1993-1994.

[B16] Filmer D, Pritchett LH (2001). Estimating wealth effects without expenditure data--or tears: an application to educational enrollments in states of India. Demography.

[B17] Bell J, Curtis SL, Alayon S (2003). Trends in Delivery Care in Six Countries. DHS Analytical Studies No. 7.

[B18] Khan MSH, Khanam ST, Nahar S, Nasreen T, Raham APMS (1999). Review of Availability and Use of Emergency Obstetric Care (EmOC) Services in Bangladesh.

[B19] Lomas J, Enkin M, Chalmers I, Enkin M, Keirse M (1989). Variations in operative delivery rates. Effective care in pregnancy and childbirth.

[B20] De Brouwere V, Van Lerberghe W (1998). Les besoins obstétricaux non-couverts.

[B21] Dubourg D, De Brouwere V, Van Lerberghe W, Richard F, Litt V, Derveeuw M (2002). The Unmet Obstetric Needs Network. Final Report. Part I Synthesis..

[B22] Dieltiens G, Dewan H, Botlero R, Alam N, Chowdhury E, Ronsmans C (2005). Pregnancy-related mortality and access to obstetric services in Matlab, Bangladesh: Tours, France..

[B23] WHO (2006). The World Health Report 2006: Working together for health.

[B24] WHO (2005). The World Health Report 2005: Make every mother and child count.

[B25] Blum LS, Sharmin T, Ronsmans C (2006). Attending home vs. clinic-based deliveries: perspectives of skilled birth attendants in Matlab, Bangladesh. Reprod Health Matters.

[B26] Afsana K (2004). The tremendous cost of seeking hospital obstetric care in Bangladesh. Reprod Health Matters.

[B27] Pitchforth E, van Teijlingen E, Graham W, Dixon-Woods M, Chowdhury M (2006). Getting women to hospital is not enough: a qualitative study of access to emergency obstetric care in Bangladesh. Qual Saf Health Care.

[B28] Chowdhury ME, Ronsmans C, Killewo J, Anwar I, Gausia K, Das-Gupta S, Blum LS, Dieltiens G, Marshall T, Saha S, Borghi J (2006). Equity in use of home-based or facility-based skilled obstetric care in rural Bangladesh: an observational study. Lancet.

[B29] Nahar S, Costello A (1998). The hidden cost of 'free' maternity care in Dhaka, Bangladesh. Health Policy Plan.

[B30] Borghi J, Ensor T, Somanathan A, Lissner C, Mills A (2006). Mobilising financial resources for maternal health. Lancet.

[B31] Hossain J, Ross SR (2006). The effect of addressing demand for as well as supply of emergency obstetric care in Dinajpur, Bangladesh. Int J Gynaecol Obstet.

[B32] Parkhurst JO, Rahman SA (2006). Life saving or money wasting? Perceptions of caesarean sections among users of services in rural Bangladesh. Health Policy.

[B33] Maine D, Akalin MZ, Chakraborty J, de Francisco A, Strong M (1996). Why did maternal mortality decline in Matlab?. Stud Fam Plann.

[B34] Houweling TAJ, Kunst AE, Mackenbach JP (2003). Measuring health inequality among children in developing countries: does the choice of the indicator of economic status matter?. International Journal for Equity in Health.

[B35] Wirth ME, Balk D, Delamonica E, Storeygard A, Sacks E, Minujin A (2006). Setting the stage for equity-sensitive monitoring of the maternal and child health Millennium Development Goals. Bull World Health Organ.

